# Journalistic Courtesy (?), the Square Deal (?) in Texas

**Published:** 1907-10

**Authors:** 


JOURNALISTIC COURTESY (?), THE SQUARE DEAL (?) IN TEXAS. —The *State Journal of Medicine* solicits the advertisements of a proprietary *not* endorsed by the great Council of Pharmacy, and disparages the *Red Back*, the *Medical News* and the *Courier Record.* I don't believe that Dr. Chase wrote this thing, for it is false, or knew or approved of it. I clip it from the *American Medical Journalist* for September. Four-fifths of my subscribers are members of the State Medical Association and many of them have been subscribers over twenty years. Please see my advertising patronage, notwithstanding this anonymous blackhander, whose libel the *State Journal of Medicine* publishes.

THE WAY TO HELP AGAINST NOSTRUMS.

The following under the above caption from the *Texas State Journal of Medicine,* affords a "living picture" of a fashion in which, among others, palpitating "proprietors" are intimidated and coerced by professional "black-handers":

"One of our readers sends a copy of a letter he writes concerns soliciting his interest in their pharmaceutcal products:

"——— *Pharmacal Company, St. Louis, Mo.*

"GENTLEMEN: Referring to your letter of the 11th inst., I fail to find Melachol among the remedies submitted to and approved by the Council on Pharmacy and Chemistry. There is no reason why a company which does not make exaggerated claims, which gives proper formulæ and which does not exploit its preparations to the laity should not receive the sanction of said council and be by it recommended to us. In no other way known to me at present can the profession be certain of the remedies which it uses.

If you desire to introduce Melachol throughout Texas, I beg to advise you that this will be the proper course to pursue. *There is but one practical way to reach the State medical profession of Texas, and that is through the State Medical Journal,* which does not receive *new* advertising which has not been approved by the Council, and this *Journal* receives the support of 60 per cent of all the legalized practitioners and 90 per cent of the reputable and active men of the State.

Assuring you of my best wishes, I beg to remain,

Yours very truly,

———"

As will be noted, independent medical journals are regarded by the *Texas State Journal's* correspondent as already exterminated and, obviously, the *Texas Medical Journal,* of Austin, *Texas Medical News,* of Dallas, and *Texas Courier-Record of Medicine,* of Fort Worth, can no longer regard themselves as of any account as a legitimate and practical means of reaching the *State* medical profession of Texas—outside of which, of course (from the same correspondent's point of view) nothing in the shape of a legitimate practitioner of medicine any longer exists. As goes without saying, what is meant by a "legitimate practioner of medicine" implies only the "union-label" variety of the "organized profession," as indicated by the "Red Cross Button" of the A. M. A. *O tempore! O mores!—American Medical Journalist,* September, 1907.

Do we understand that "the way to help against nostrums" is to get them to advertise in the *State Journal of Medicine?* It reads that way.

